# Successful treatment with proton beam therapy for a solitary sternal metastasis of breast cancer: a case report

**DOI:** 10.1186/s13256-022-03335-5

**Published:** 2022-03-20

**Authors:** Yojiro Ishikawa, Motohisa Suzuki, Hisashi Yamaguchi, Ichiro Seto, Masanori Machida, Yoshiaki Takagawa, Keiichi Jingu, Yasuyuki Kikuchi, Masao Murakami

**Affiliations:** 1 Department of Radiation Oncology, Southern Tohoku Proton Therapy Center, 7-172, Yatsuyamada, Koriyama, Fukushima 963-8052 Japan; 2 Department of Radiation Oncology, Tohoku University Graduate School of Medicine, 1-1, Seiryo-chou, Aoba-ku, Sendai, Miyagi 980-8574 Japan

**Keywords:** Proton beam therapy, Breast cancer, Sternal metastasis

## Abstract

**Background:**

Breast cancer infrequently metastasizes to the sternum as solitary metastasis. We experienced successful treatment with proton beam therapy for a case of sternal metastasis of breast cancer. This case demonstrates for the first time the role of proton therapy in the treatment of oligometastatic sternal metastasis with limited tolerance of normal tissue due to previous photon irradiation.

**Case presentation:**

A 40-year-old Japanese female presented with lumpiness in her left breast. The patient was diagnosed with breast cancer (cT1N0M0, cStage IA) and underwent partial mastectomy with axillary lymph node dissection. After the mastectomy, the patient received radiation therapy with 50 Gy in 25 fractions for initial irradiation of the left breast. After the initial irradiation of 50 Gy, the patient received 10 Gy in five fractions of a sequential boost for the tumor bed to a total dose of 60 Gy. Although the patient was administered tamoxifen after radiation therapy, solitary sternal metastasis occurred 6 months after radiation therapy. She refused chemotherapy and requested proton beam therapy for her sternal metastasis. The daily proton beam therapy fractions were 2.5 relative biological effectiveness, receiving a total dose of 70 Gy relative biological effectiveness. An acute side effect of grade 2 dermatitis according to the National Cancer Institute Common Terminology Criteria for Adverse Events version 4.0. occurred during proton beam therapy, but there was no acute or late complication of more than grade 3. At 3 years after proton beam therapy, the patient remains in complete remission without surgery or chemotherapy.

**Discussion and conclusion:**

Proton beam therapy for solitary sternal metastasis of breast cancer is considered to be a therapeutic option.

## Background

Bone metastasis of breast cancer generally tends to be multiple. However, breast cancer rarely metastasizes to the sternum as solitary metastasis [1]. Management of metastatic breast cancer (MBC) is based on systemic treatment, while the role of local therapy remains controversial.

Proton beam therapy (PBT) is effective because protons have excellent dose localization according to the Bragg peak compared with photons and are biologically equivalent to conventional X-ray treatment for cancer [2–5]. In general, bone metastases are multiple metastases and are not an indication for PBT. We herein report the achievement of successful treatment with proton beam therapy for a solitary sternal metastasis of breast cancer. This case demonstrates for the first time the role of proton therapy in the treatment of oligometastatic sternal metastasis with limited tolerance of normal tissue due to previous photon irradiation.

## Case presentation

**Fig. 1:**
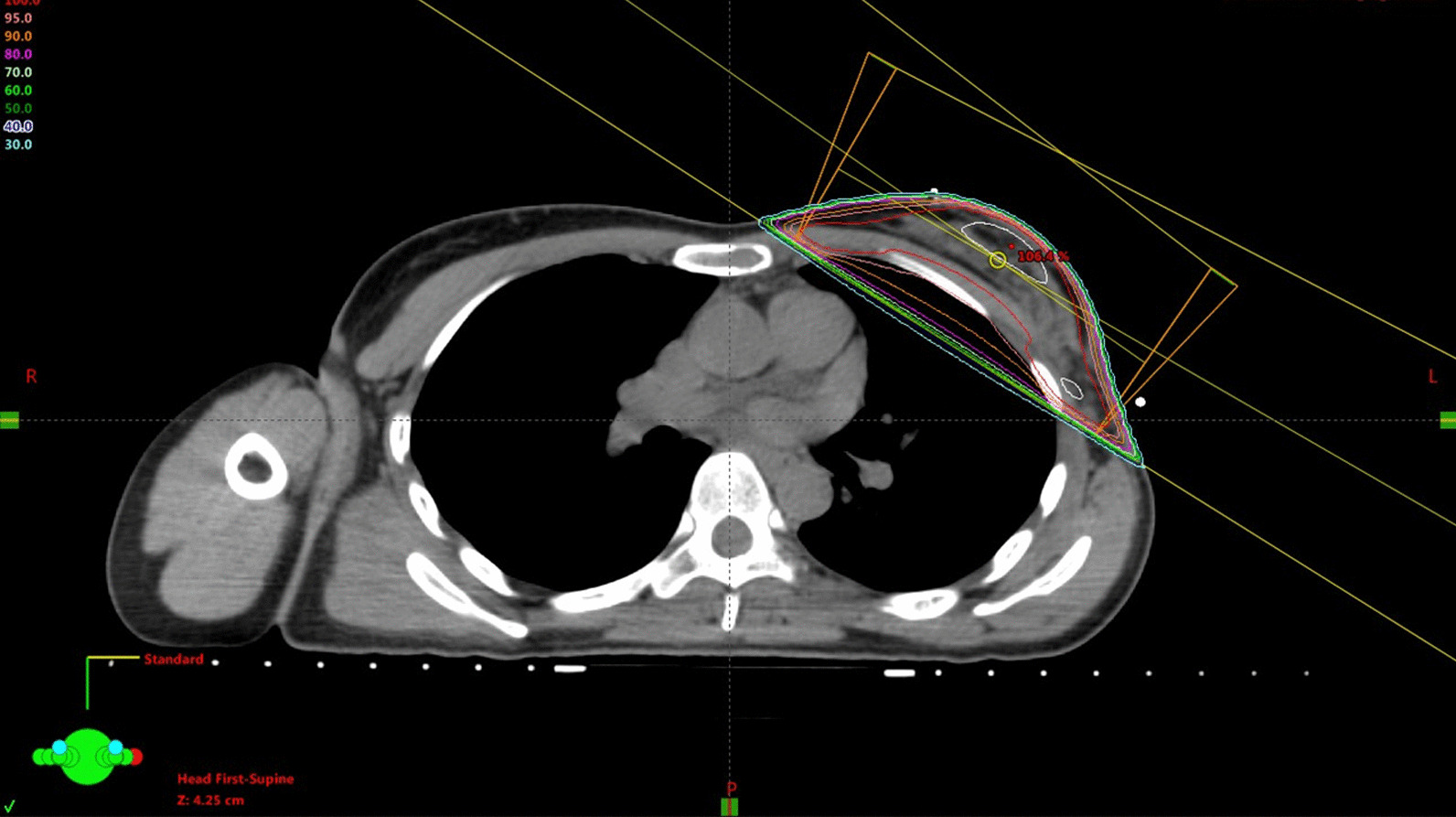
Dose distribution of radiation therapy. The left breast after partial mastectomy was treated with 50 Gy

One year before presentation to our hospital, a 40-year-old Japanese female presented to another hospital with lumpiness in her left breast. The patient had no medical history or family history of breast or ovary cancer. There was no history of drinking or smoking. At the previous hospital, a core needle biopsy revealed invasive ductal carcinoma, estrogen receptor-positive, progesterone receptor-positive, and human epidermal growth factor receptor 2-negative with a Ki-67 index of 23%. On the basis of the examination results, the patient was diagnosed with early breast cancer (cT1N0M0, cStage I) and underwent partial mastectomy with axillary lymph node dissection. After the mastectomy, the patient received radiation therapy (RT) with 50 Gray (Gy) in 25 fractions as initial irradiation for the left breast. After the initial irradiation of 50 Gy, she received 10 Gy in five fractions of a sequential boost for the tumor bed to a total dose of 60 Gy (Fig. 1). Tamoxifen was administered after RT.

**Fig. 2:**
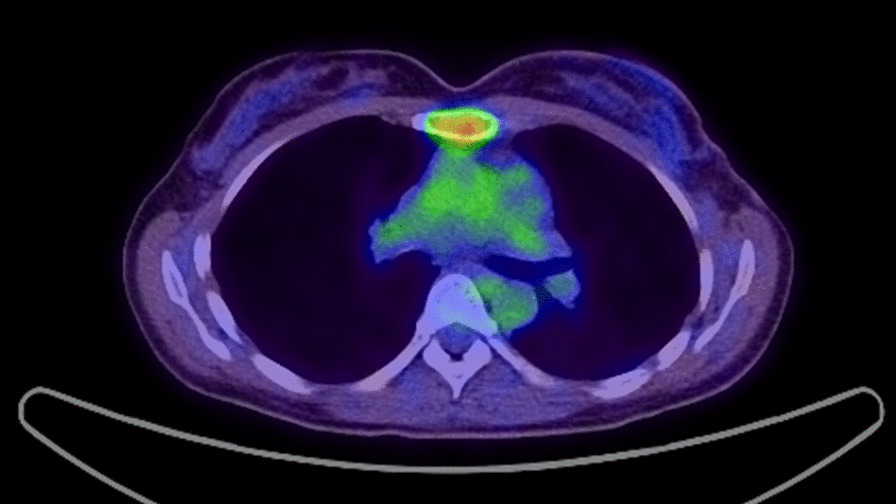
Positron emission tomography–CT revealed uptake of 18F-2-fluoro-2-deoxy-d-glucose in the sternum (maximum standardized uptake value of 4.5)

At 6 months after RT, she presented with a dull pain in her chest wall. Positron emission tomography–computed tomography (PET–CT) revealed uptake of 18F-2-fluoro-2-deoxy-d-glucose (FDG) in the sternum [maximum standardized uptake value (SUV_max_) of 4.5] (Fig. 2). Because the patient had a history of breast cancer and FDG-PET results showed a low possibility of malignant disease other than breast cancer, biopsy examination was omitted. The diagnosis was sternal metastasis of breast cancer. The patient had an Eastern Cooperative Oncology Group Performance Score of 1.

Although the patient was recommended chemotherapy and RT for bone metastasis of the sternum, she refused to receive chemotherapy and RT owing to concerns about damage caused by these therapies. She and her family requested PBT for her bone metastasis of the sternum. Treatment with the aromatase inhibitor and luteinizing-hormone-releasing hormone agonists was also started at the previous hospital.

The patient was informed of the option of receiving PBT as an alternative to RT. It was difficult to use definitive RT of more than 60 Gy for the metastasis because the sternal metastasis was located close to the initial field of RT. We also considered stereotactic radiotherapy or volumetric modulated arc therapy for her bone metastasis. However, we did not select these therapies because of the increased risk of radiation-induced cardiotoxicity by X-ray treatment.

**Fig. 3:**
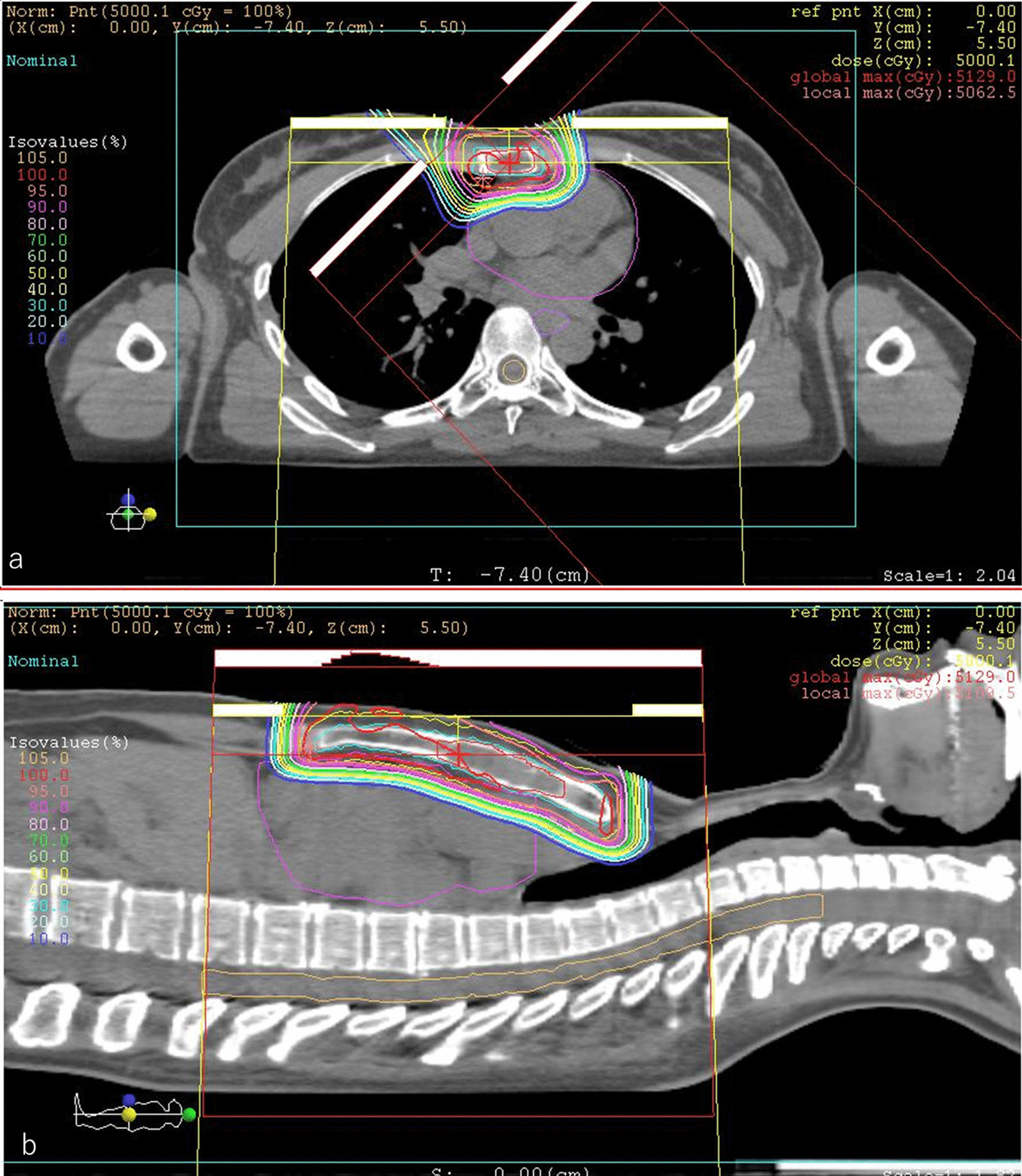
Dose distribution of initial proton beam therapy (PBT) in an axial field (**a**) and a coronal field (**b**). The daily PBT fractions were 2.5 relative biological effectiveness (RBE) for PTV, receiving a total dose of 50 Gy RBE. The gross tumor volume was 16.21 cm^3^. The clinical target volume was 60.44 cm^3^, and the planning target volume was 147.31 cm^3^

**Fig. 4:**
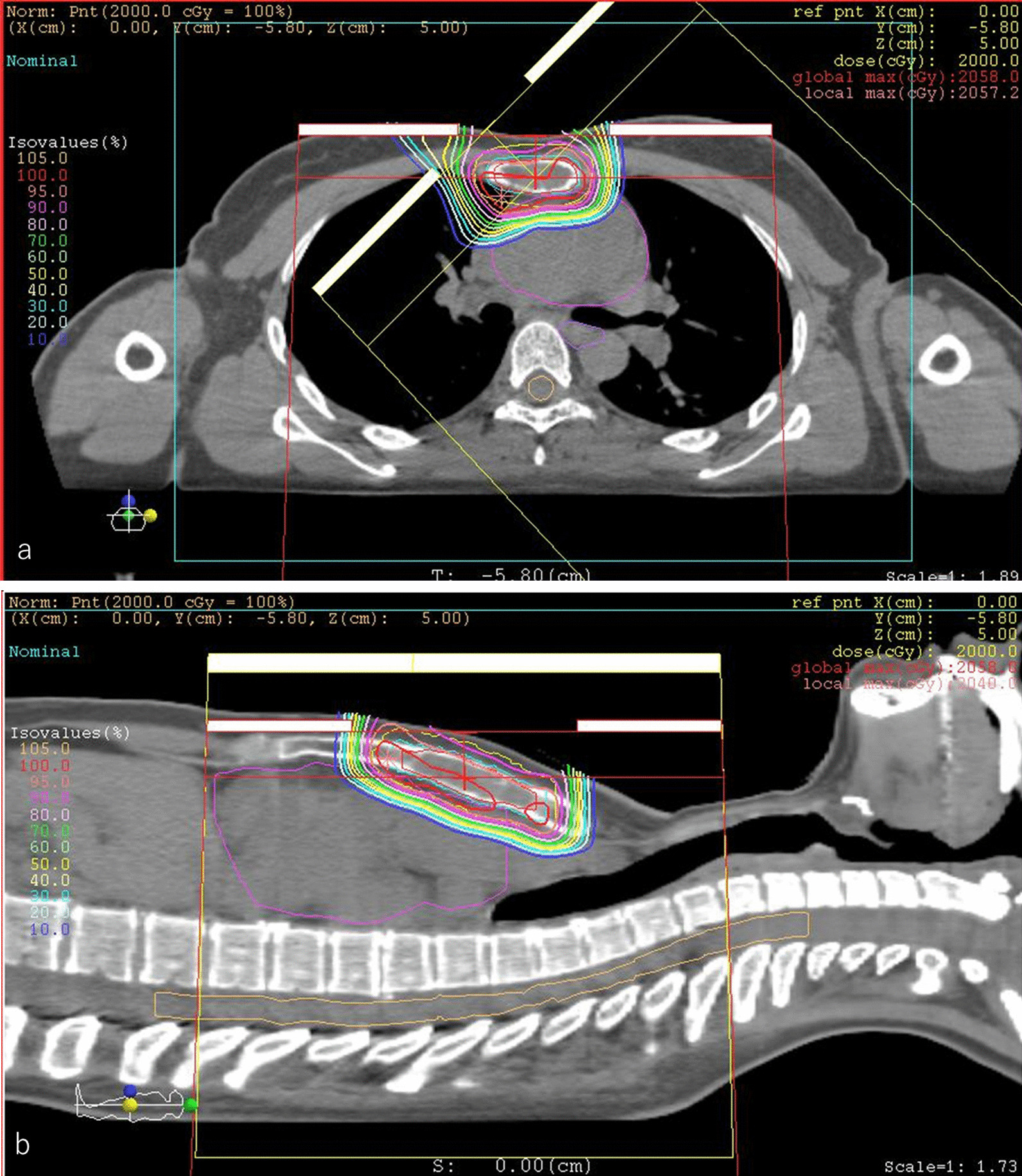
Dose distribution of boost proton beam therapy in an axial field (**a**) and a coronal field (**b**). The patient received 20 Gy relative biological effectiveness (RBE) in five fractions of a sequential boost for the sternal metastasis alone to a total dose of 70 Gy RBE. The gross tumor volume was 16.21 cm^3^. The clinical target volume was 29.89 cm^3^, and the planning target volume was 70.96 cm^3^

**Fig. 5:**
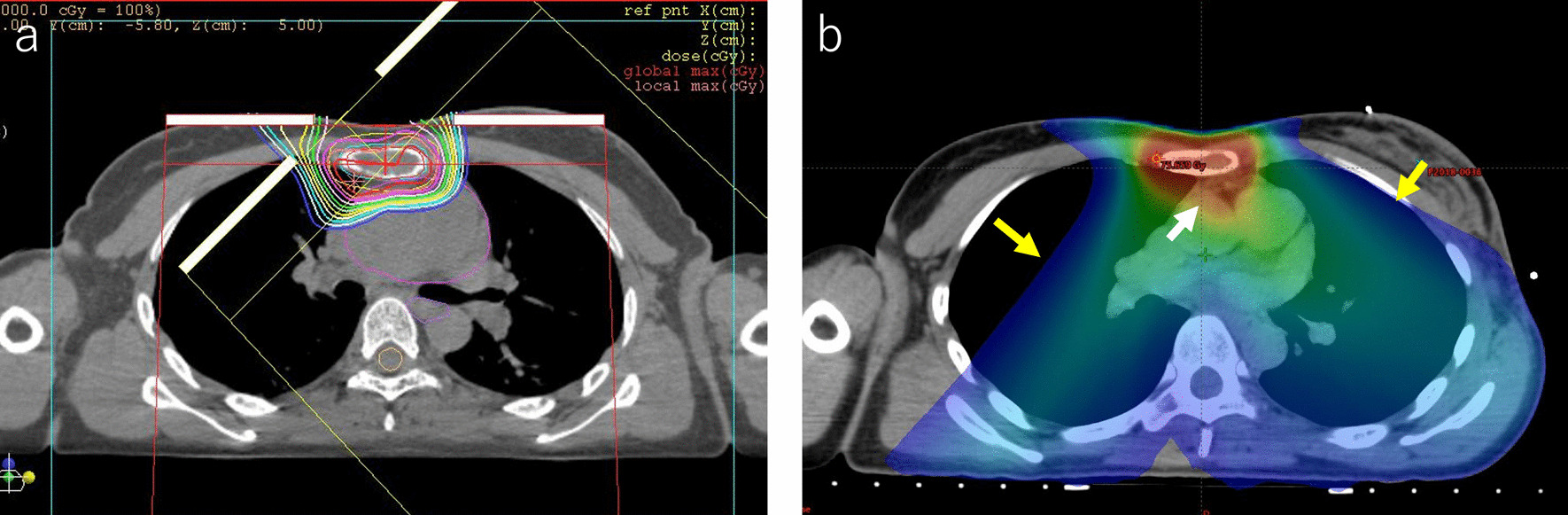
Dose distribution for proton beam therapy (**a**) and photon beam therapy (**b**). Photon beam therapy shows a dose distribution of 70 Gy in 28 fractions by intensity-modulated radiation therapy (IMRT). With the IMRT, a high-dose area was seen on the heart (white arrow), and a low-dose area was spread to the bilateral lungs (yellow arrow)

We gave the patient an explanation about late complications after PBT. She agreed to receive PBT in favor of tumor control despite late complications. The PBT system at our institute (Proton beam system, Mitsubishi, Tokyo, Japan) uses synchrotron and scattering methods. The gross tumor volume (GTV) included the bone metastasis of the sternum. The clinical target volume (CTV) was defined as GTV plus 0.5-cm margins and the whole sternum. We determined the CTV on the basis of the margins that surgery is performed on a solitary sternal metastasis. The planning target volume (PTV) was CTV plus 0.5-cm margins. The daily PBT fractions were 2.5 relative biological effectiveness (RBE) for PTV that received a total dose of 70 Gy RBE in 28 fractions. The overall treatment duration was 41 days. After initial irradiation of 50 Gy RBE in 20 fractions (Fig. 3), the patient received 20 Gy RBE in 8 fractions of a sequential boost for the sternal metastasis alone to a total dose of 70 Gy RBE (Fig. 4). The dosimetric comparison between PBT and photon beam therapy is shown in Table 1 and Figure 5.

**Table 1:** Dosimetric comparison between proton beam therapy and photon beam therapy

OAR	DVH parameter	PBT	IMRT (VMAT)
Lung	V20 (%)	2.02	21.98
	V5 (%)	3.91	66.6
	Mean dose (Gy)	1.15	12.3
Heart	V40 (%)	4.39	7.8
	V30 (%)	5.75	19.7
	V20 (%)	7.33	44.1
	Mean dose (Gy)	3.92	18.65
Esophagus	Maximum dose (Gy)	0.84	32.5
Spinal cord	Maximum dose (Gy)	0	18.643

*DVH* dose–volume histogram; *IMRT* intensity-modulated radiation therapy; *OAR* organ at risk; *PBT* proton beam therapy; *VMAT* volumetric modulated arc therapy

**Fig. 6:**
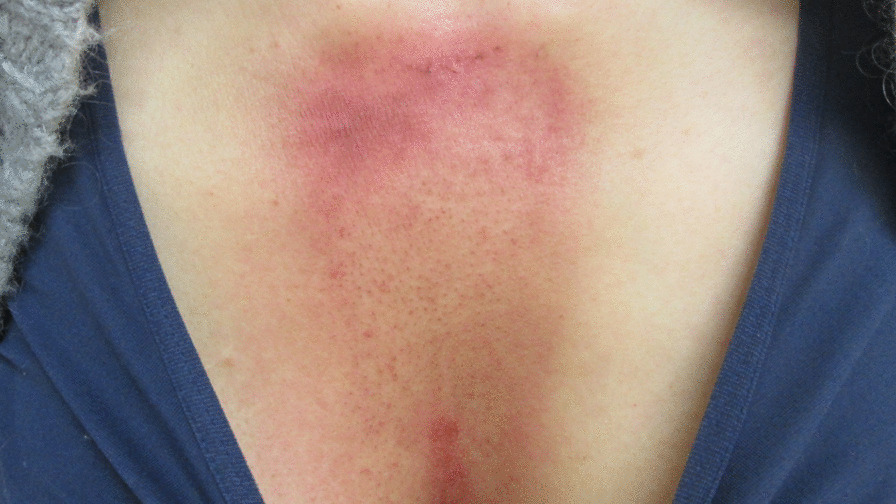
Macroscopic findings of the chest on the final day of proton beam therapy. The acute side effect in skin was grade 2 dermatitis according to the National Cancer Institute Common Terminology Criteria for Adverse Events version 4.0

The acute side effect of grade 2 dermatitis according to the National Cancer Institute Common Terminology Criteria for Adverse Events version 4.0. occurred during PBT (Fig. 6), but there was no acute or late complication higher than grade 3. Four months after PBT, the patient became aware of pain near the radiation field after exercising.

**Fig. 7:**
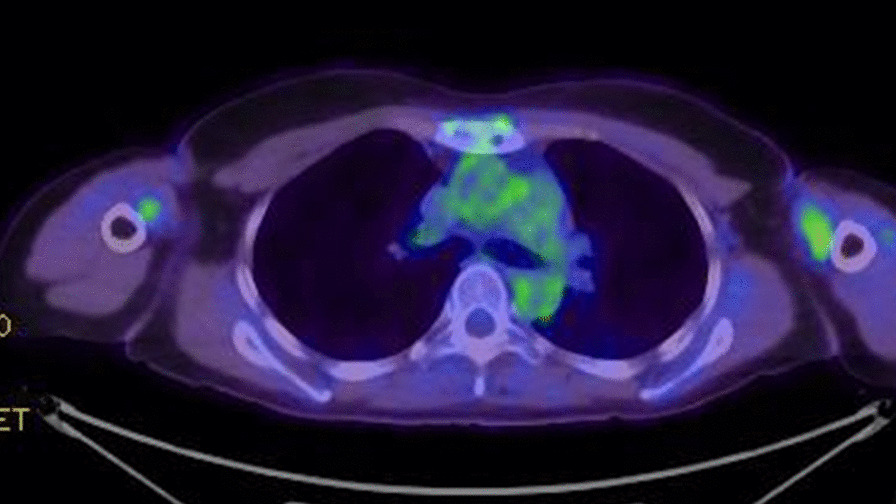
Positron emission tomography 3 years after proton beam therapy (PBT). PBT resulted in the disappearance of high uptake of fluorodeoxyglucose in the sternum

Although the symptoms resolved in about a month, the hormone therapy was continued. At 3 years after PBT, the patient remains in complete remission without surgery or chemotherapy (Fig. 7).

**Fig. 8:**
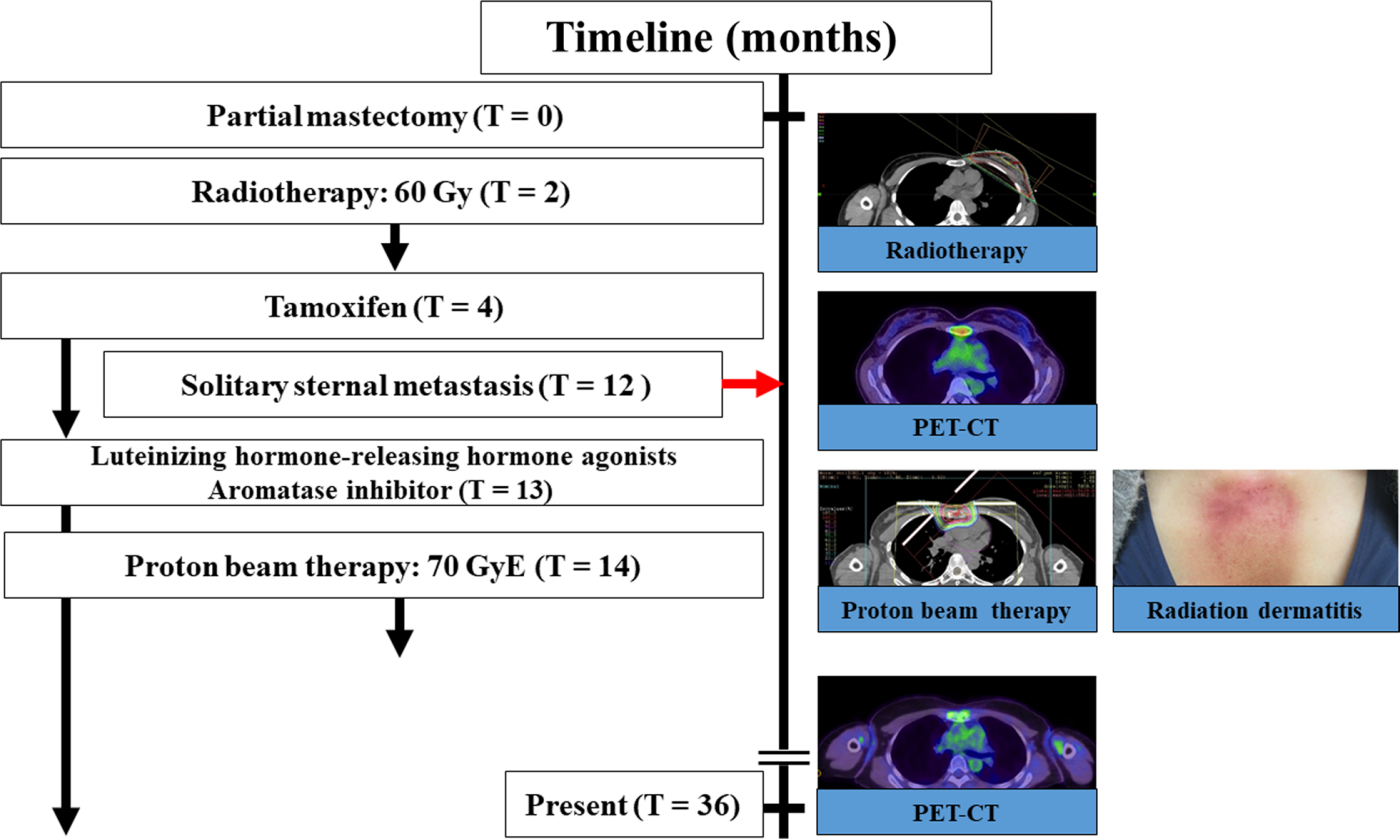
Timeline for intervention and clinical outcome

The timeline for the present case is shown in Fig. 8.

## Discussion

Metastatic breast cancer is difficult to cure. The main treatment for MBC is systemic therapy, and the role of local therapy remains controversial. There are rare cases where only a few metastases occur in MBC. Bone metastases are the most common metastases of breast cancer. Patients with solitary bone metastasis account for approximately half of all breast cancer patients [6]. Sternal metastasis from breast cancer can occur as a result of direct spread from involved intramammary nodes or isolated intramanubrial bone metastases [7]. Kozumi *et al.* reported that the incidence of metastasis in the sternum was 34% in patients with solitary bone metastasis [1]. Solitary sternal metastasis can remain solitary and confined to the sternum for a long time because the blood in the sternum lacks communication to the paravertebral venous plexus flow [8, 9].

Cancer status with fewer than five metastatic or recurrent lesions and with controlled primary lesions can be considered as “oligo-metastases” or “oligo-recurrence” [10]. The term “oligo-metastases” was first reported in 1995 by Hellman and Weichselbaum [11]. Several retrospective studies showed that patients with oligo-metastases who received local therapy had a good prognosis. The overall survival rate in those patients was 82% at 10 years and 53% at 20 years [12]. It is reasonable to consider that our case was oligo-recurrence of a solitary sternal metastasis with a good prognosis.

In current guidelines for and reports on palliative radiation therapy for patients with breast cancer, a single fraction of 8–10 Gy or 20 Gy in 4–5 fractions is recommended for patients with poor performance status (PS), and 30 Gy in 10 fractions or 50 Gy in 25 fractions is recommended for patients with good PS [13–15].

It is difficult to cure bone metastases with radiation therapy at doses less than 50 Gy. Li *et al.* reported that a single fraction of RT with 20 Gy for sternal metastases in oligometastatic breast cancer was beneficial for local disease control. In their study, in-field control was achieved in nine of ten patients at a median follow-up of 32 months; however, they also reported that seven of the ten patients had distant relapse after RT, and the median time to distant relapse was 11 months. Kamiyoshihara *et al.* also reported a severe complication after radiation therapy for sternal metastasis. Although there was no detailed information about the radiation therapy in their report, aortic hemorrhage due to mediastinal infection occurred in one patient after irradiation for sternal metastasis [16].

We experienced successful treatment with PBT for a case of sternal metastasis of breast cancer. It is not surprising that bone metastasis is controlled well locally by PBT; however, no case similar to our case was found in the English literature in a PubMed search (available at http://www.ncbi.nlm.nih.gov/pubmed/) using “breast cancer” and “sternal metastasis” connected with “proton beam therapy” as index words. This is the first report of successful treatment with proton beam therapy for sternal metastasis from breast cancer.

## Conclusion

Because this study was a case study, it is difficult to define the indication for PBT for solitary sternal metastasis of breast cancer. However, it is possible that some patients with solitary sternal metastasis of breast cancer were treated only by surgery, chemotherapy, or radiation therapy despite being potential candidates for PBT. PBT for solitary sternal metastasis of breast cancer is considered to be a therapeutic option.

## Data Availability

The data include individual patient data, but the data are available from the corresponding authors upon reasonable request.

## Abbreviations

FDG: 18F-2-fluoro-2-deoxy-d-glucose
Gy: Gray
IMRT: Intensity-modulated radiation therapy
MBC: Management of metastatic breast cancer
MRI: Magnetic resonance imaging
PBT: Proton beam therapy
PET–CT: Positron emission tomography–computed tomography
PS: Performance status
RT: Radiation therapy
RBE: Relative biological effectiveness
SUV_max_: Maximum standardized uptake value
VMAT: Volumetric modulated arc therapy

## References

[1] Koizumi M, Yoshimoto M, Kasumi F, Ogata E. Comparison between solitary and multiple skeletal metastatic lesions of breast cancer patients. Ann Oncol. 2003;14:1234–40. 10.1093/annonc/mdg348. 12881385

[2] Makishima H, Ishikawa H, Terunuma T, *et al*. Comparison of adverse effects of proton and X-ray chemoradiotherapy for esophageal cancer using an adaptive dose–volume histogram analysis. J Radiat Res. 2014;56:568–76. 10.1093/jrr/rrv001. PMC4426925 25755255

[3] Hirano Y, Onozawa M, Hojo H, *et al*. Dosimetric comparison between proton beam therapy and photon radiation therapy for locally advanced esophageal squamous cell carcinoma. Radiat Oncol. 2018;13:1–9. 10.1186/s13014-018-0966-5. PMC5807768 29426342

[4] Williamson JF. Physics contribution. Int J Radiat Oncol Biol Phys. 1998;41:959–70. 10.1016/s0360-3016(98)00150-3 9652865

[5] Zhang X, le Zhao K, Guerrero TM, *et al*. Four-dimensional computed tomography-based treatment planning for intensity-modulated radiation therapy and proton therapy for distal esophageal cancer. Int J Radiat Oncol Biol Phys. 2008;72:278–87. 10.1016/j.ijrobp.2008.05.014. PMC2610812 18722278

[6] Soni A, Ren Z, Hameed O, *et al*. Breast cancer subtypes predispose the site of distant metastases. Am J Clin Pathol. 2015;143:471–8. 10.1309/AJCPYO5FSV3UPEXS. 25779997

[7] Motono N, Shimada K, Kamata T, Uramoto H. Sternal resection and reconstruction for metastasis due to breast cancer: the Marlex sandwich technique and implantation of a pedicled latissimus dorsi musculocutaneous flap. J Cardiothorac Surg. 2019;14:79. 10.1186/s13019-019-0905-z. PMC6471832 30999925

[8] Noguchi S, Miyauchi K, Nishizawa Y, *et al*. Results of surgical treatment for sternal metastasis of breast cancer. Cancer. 1988;62:1397–401. 10.1002/1097-0142(19881001)62:7<1397::aid-cncr2820620726>3.0.co;2-2. 3416279

[9] Demetrian AD, Olteanu M, Mîndrilă I, *et al*. Long disease-free survival following total sternal resection and reconstruction of the sternum with acrylic cement for unique massive sternal metastasis after operated breast cancer. Roman J Morphol Embryol. 2018;59:1225–32. 30845305

[10] Niibe Y, Hayakawa K. Oligometastases and oligo-recurrence: the new era of cancer therapy. Jpn J Clin Oncol. 2010;40:107–11. 10.1093/jjco/hyp167. PMC2813545 20047860

[11] Hellman S, Weichselbaum RR. Oligometastases. J Clin Oncol. 1995;13:8–10. 10.1200/JCO.1995.13.1.8 7799047

[12] Coombe R, Lisy K, Campbell J, *et al*. Survival outcomes following aggressive treatment of oligometastatic breast cancer: a systematic review protocol. JBI Database Syst Rev Implement Rep. 2017;15:2013–9. 10.11124/JBISRIR-2016-002954. 28800049

[13] Lutz S, Balboni T, Jones J, *et al*. Palliative radiation therapy for bone metastases: update of an ASTRO evidence-based guideline. PRRO. 2017;7:4–12. 10.1016/j.prro.2016.08.001. 27663933

[14] Gradishar WJ, Anderson BO, Abraham J, *et al.* NCCN Guidelines (Version 5.2020): Invasive Breast Cancer. 67 (2020)

[15] Jacobson G, Kaidar-Person O, Haisraely O, *et al*. Palliative radiation therapy for symptomatic advance breast cancer. Sci Rep. 2021;11:5282. 10.1038/s41598-021-84872-9. PMC7970854 33674709

[16] Kamiyoshihara M, Ibe T, Igai H, *et al*. Profuse mediastinal hemorrhage due to mediastinitis after a sternal infection. Ann Thorac Cardiovasc Surg. 2014;20(Suppl):629–31. 10.5761/atcs.cr.13-00010. 23995346

